# Post COVID-19 Symptoms Among Infected Vaccinated Individuals: A Cross-Sectional Study in Saudi Arabia

**DOI:** 10.1007/s44197-023-00146-9

**Published:** 2023-09-04

**Authors:** Heba M. Adly, Saleh A. K. Saleh, Mohammed A. Garout, Altaf A. Abdulkhaliq, Abdullah A. Khafagy, Abdullah A. Saati, Imad A. AlJahdali, Maher N. Alandiyjany, Jaffar A. Al-Tawfiq

**Affiliations:** 1 Community Medicine and Pilgrims Healthcare Department, Faculty of Medicine, Umm Al-Qura University, Makkah, Saudi Arabia; 2 Biochemisty Department, Faculty of Medicine, Umm Al-Qura University, Makkah, Saudi Arabia; 3 Oncology Diagnostic Unit, Faculty of Medicine, Ain Shams University, Cairo, Egypt; 4 Laboratory Medicine Department, Faculty of Applied Medical Sciences, Umm Al-Qura University, 21955 Makkah, Saudi Arabia; 5 Quality and Development Affair, Batterjee Medical College, 21442 Jeddah, Saudi Arabia; 6 Specialty Internal Medicine and Quality Department, Johns Hopkins Aramco Healthcare, Dhahran, Saudi Arabia; 7 Infectious Diseases Division, Department of Medicine, Indiana University School of Medicine, Indianapolis, IN USA; 8 Infectious Diseases Division, Department of Medicine, Johns Hopkins University School of Medicine, Baltimore, MD USA

**Keywords:** COVID-19, Pandemic, Infection, Coronavirus, Post recovery, Symptoms, Saudi Arabia, SARS-CoV-2

## Abstract

**Introduction:**

Multiple studies investigated the endurance and occurrence of symptoms three months after SARS-CoV-2 infection. This study examines the possible effects of COVID-19 vaccination on the persistence of post-recovery symptoms.

**Patients and Methods:**

A cross-sectional survey was conducted in Saudi Arabia to evaluate 14 prevalent long COVID-19 symptoms among vaccinated individuals. Patients self-reported their acute COVID-19 experience, demographic information, chronic conditions, vaccine history, and persistent symptoms.

**Results:**

Of the 484 patients, four respondents were excluded from the study as they had not received the vaccine, and 111 (23.1%) were vaccinated but did not get infected and were also excluded. The remaining 369 (76.9%) reported COVID-19 and a vaccination and thus they were included in the study. The occurrence of post-COVID-19 symptoms was reported in 59 (16.1%) for ≤ 3 months, 202 (54.8%) experienced persistent symptoms 3–6 months, and 108 (29.1%) reported symptoms lasting > 6 months. In relation to age group, persistent symptoms 3–6 months after recovery was more common in those > 50 years and symptoms lasting > 6 months were more common in 30–50 years of age (p < 0.001). Persistence of symptoms for  3-6 months was more common in those who were infected prior to vaccination compared to those who were infected after vaccination (P < 0.001). Of the included patients, 323 (87.5%) rated their health as good, 41 (11.1%) considered it fair, and 5 (1.4%) described their well-being as poor or terrible.

**Conclusion:**

The study provides information of persistent symptoms in vaccinated individuals who had recovered from COVID-19 and highlights the need for targeted interventions to alleviate post-COVID-19 symptoms. The study is limited by its reliance on self-reported data and potential selection bias. Future research is needed to understand the mechanisms underlying persistent symptoms in vaccinated individuals and to identify effective interventions for long COVID.

## Introduction

Severe acute respiratory syndrome coronavirus 2 (SARS-CoV-2) is responsible for the global pandemic of Coronavirus Disease-19 (COVID-19), declared by the World Health Organization on March 11, 2020 [1]. As of April 2023, approximately 68.4% of the global population has received at least one dose of a COVID-19 vaccine [2]. While the daily vaccination rate worldwide is around 4.25 million, only 24% of individuals in low-income countries have received a minimum of one dose [3].

COVID-19 vaccines have demonstrated effectiveness in preventing severe illness, hospitalization, and death [4]. Vaccines also significantly reduce the likelihood of hospitalization, and vaccinated individuals who do contract SARS-CoV-2 tend to experience milder symptoms compared to the unvaccinated [5]. In one study, vaccination has been shown to decrease the severity and long-term impact of persistent symptoms, commonly known as long COVID, at 120 days [6]. Long COVID, or the persistence of symptoms following acute SARS-CoV-2 infection, is increasingly recognized [7]. Common symptoms include fatigue, breathlessness, myalgia, and insomnia [8]. Studies conducted in the UK on participants aged 18 to 69 years showed that the first COVID-19 vaccination reduced the likelihood of self-reported long COVID by 13%, and the second dose further decreased the likelihood by 9% [9].

The prevalence of long COVID after SARS-CoV-2 infection has been reported to be six times higher than post-other viral diseases, ranging from 9 to 63% [10]. Another study showed that the prevalence of symptoms varies from 35% to 90.5% [11]. A previous study conducted in Saudi Arabia between May and June 2021 reported that approximately half of the surveyed COVID-19 patients experienced post-COVID symptoms. However, the study did not consider the impact of COVID-19 vaccination on reducing long COVID symptoms [12]. Another study demonstrated that the rate of post-COVID-19 symptoms decreased as the number of vaccine doses increased. The rates of the condition were 41.8% (95% CI, 37.0%-46.7%), 30.0% (95% CI, 6.7%-65.2%), 17.4% (95% CI, 7.8%-31.4%), and 16.0% (95% CI, 11.8%-21.0%) for individuals who received 0, 1, 2, and 3 doses of the vaccine, respectively [13]. In this study, we aim to evaluate the occurrence and types of long COVID-19 among vaccinated individuals who had infection.

## Patients and Methods

### Study Design

In this study, we examined the effects of various vaccines on the occurence of 14 post-COVID-19 symptoms to identify potential patterns. The questionnaire was circulated through WhatsApp groups, including those for healthcare professionals and the general public, between August and October 2022. Post-COVID-19 symptoms refer to a range of symptoms persisting or developing three months following infection and lasting at least two months with no other explanation [14]. Specifically, symptoms may begin as early as one month following infection and can last for an extended period, with some patients experiencing symptoms beyond six months. We distinguished between different stages of persistence: short-term (< 3 month), medium-term (3–6 months), and long-term (lasting more than six months). We limited the survey to patients who tested positive via PCR or antigen tests, and thus confirming positive cases. The collected data included the type and timing of the vaccine received, the date and severity of the initial illness, pre-existing chronic conditions, and demographic information. Before participating, respondents were asked to consent to their involvement in the study. The study protocol was approved by the Ethics Review Board for Human Studies at the Faculty of Medicine, Umm Al-Qura University (Approval no. HAPO-02-K-012–2022-09–1180), in compliance with the guidelines set forth by the Saudi National Committee for Bioethics (HABO-02-K-012).

The survey collected essential data, such as age, gender, nationality (Saudi and non-Saudi citizens), area of residence, chronic disease status, hospitalization history (severity of illness, length of hospital stays, need for respiratory support, and requirement for intensive care unit [ICU]), duration since symptom onset, and persistence of symptoms. Patients self-reported information regarding their acute COVID-19 experience. The severity of the disease can be mild to moderate, severe, or critical. Patient symptoms were grouped by system: general (fatigue, myalgia), respiratory (chest pain, cough, wheezing), cardiovascular (palpitations), neuropsychiatric (headache, hypersomnia, depression, anxiety), dermatological (hair loss), and gastrointestinal (diarrhea, constipation). The severity of symptoms was determined based on the patients’ reported outcomes and graded from none to severe. The study considered various COVID-19 vaccines, including Pfizer/BioNTech (BNT162b2), Oxford-AstraZeneca (ChAdOx1 nCoV-19), Moderna (mRNA-1273), and Janssen (Johnson & Johnson; JNJ-78436735 or Ad26.COV2.S). Vaccination status and vaccine doses either before or after SARS-CoV-2 infection were also obtained.

### Statistical Analysis

All analyses were conducted using the SPSS software (version 21). Socio-demographic characteristics of the study sample were expressed as total percentages, means, and standard deviations. Descriptive statistics were reported as mean ± standard deviation (SD) or medians along with quartiles (25th–75th percentile) for continuous variables that lacked a normal distribution, and as frequency and percentages for categorical variables. A t-test was utilized to assess continuous variables with normally distributed data. A p-value below 0.05 was considered statistically significant.

## Results

### Patients Demographic Characteristics

**Fig. 1:**
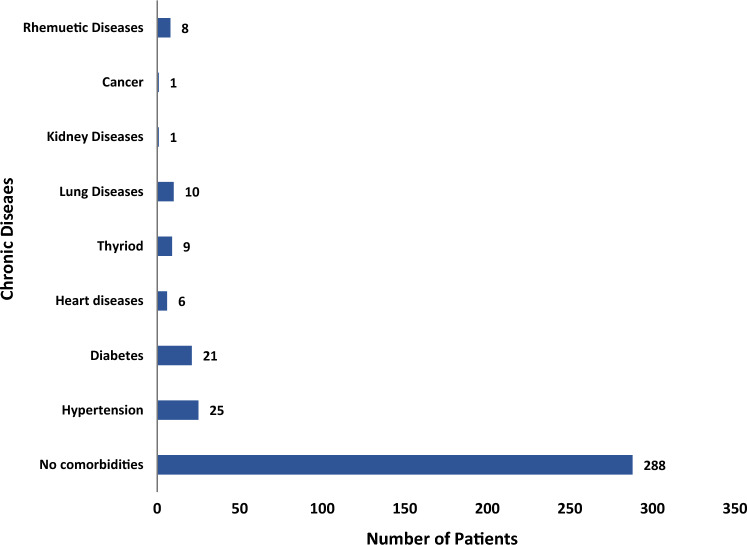
Presence or absence of underlying comorbid diseases among the included patients

A total of 484 individuals participated in the study through an online questionnaire circulated among various WhatsApp groups. All patients had recovered from COVID-19 and tested positive via PCR or antigent test. Four respondents were excluded from the study as they had not received the vaccine, and 111 (23.1%) were vaccinated but did not get infected and were also excluded. The remaining 369 (76.9%) reported COVID-19 and a vaccination and were included in the final analysis. Among the 369, 192 (52%) were female and 177 (48%) were male; and there were 206 (55.8%) Saudis and 163 (44.2%) non-Saudis (Table 1). There were 232 (63%) healthcare workers, 130 (35.4%) were employed in other professions, and 7 (1.6%) were not working. Of the included patients, 288 (76%) reported no comorbidities, and 81 (24%) had at least one comorbidity such as hypertension (10.5%), diabetes mellitus (6.7%), and heart diseases (1%) (Fig. 1).

**Table 1:** Demographic and general attributes of infected vaccinated individuals investigated for post COVID-19 symptoms among (n = 369)

Attributes of patients	Value
Age, years, n (%)	18–29, 70 (19%)
	30–50, 233 (63%)
	≥ 50, 66 (18%)
Gender	Males 177 (48%)
	Females 192 (52%)
Nationality	Saudi, 206 (55.8%)
	Non-Saudi, 163 (44.2%)
Residence area	Western region, 258 (70%)
	Eastern region, 75 (20.3%)
	Southern region, 19 (5.2%)
	Middle region, 17 (4.5%)
Participant profession	Healthcare workers, 232 (63%)
	Different profession, 130 (35.4%)
	No work, 7 (1.6%)
Comorbidities	
No comorbidity	288 (76%)
At least one comorbidity	81 (24%)

Of the included patients, 70 (19%) were 18–29 years old, 233 (63%) were 30–50 years old, and 66 (18%) were ≥ 50 years old. In a multivariate analysis, co-morbidity status showed a significant difference across the age groups (p < 0.001) (Table 2). Most of patients without co-morbidities were in the 30–50 age group (70.5%), while most (53.1%) patients with one or more co-morbidities were over 50 years of age. Vaccination type and dosage revealed significant differences across age groups (p < 0.05). For Pfizer (BNT162b2) and Oxford vaccines (ChAdOx1 nCoV-19), the largest percentage of patients receiving first, second, and booster doses were in the 30–50 age group. In relation to age group, persistent symptoms 3–6 months after recovery was more common in those > 50 years and symptoms lasting > 6 months were more common in 30–50 years of age (p < 0.001) (Table 2).

**Table 2:** Multivariate analysis comparing three age groups (18–29 years; 30–50 years; and ≥ 50 years)

Patients characteristics	Age group in years				*P* value
	Total n = 369	18–29 years n = 70	30–50 years n = 233	≥ 50 years n = 66	
Gender, n (%)					
Males, n (%)	177	27 (38.6%)	101 (43.3%)	49 (74.2%)	< 0.001
Females, n (%)	192	43 61.4%)	132 (56.7%)	17 (25.8%)	< 0.001
Nationality					
Saudi, n (%)	206	66 94.3%)	113 (48.5%)	27 (47.4%)	< 0.001
Non-Saudi, n (%)	163	4 (5.7%)	120 (51.5%)	39 (52.6%)	
Profession					
Healthcare workers	232	48 (68.6%)	143 (61.4%)	41 (71.9%)	< 0.001
Non-healthcare workers	130	22 (31.4%)	87 (37.3%)	21 (36.8%)	
Not working	7	0 (0)	3 (1.3%)	4 (7%)	
Co-morbidities					
No–co morbidity	288	62 (88.6%)	203 (87.1%)	23 (40.4%)	< 0.001
≥ One co-morbidity	81	8 (11.4%)	30 (12.9%)	43 (75.4%)	
Vaccine type					
Pfizer vaccine (BNT162b2)					
1st dose	209	55 (78.6%)	120 (51.5%)	34 (51.5%)	< 0.05
2nd dose	213	45 (64.3%)	135 (58%)	33 (50%)	
Booster dose	274	44 (62.9%)	182 (78.1%)	48 (72.7%)	
Oxford vaccine (ChAdOx1 nCoV-19)					
1st dose	94	11 (15.7%)	65 (27.9%)	18 (27.3%)	< 0.05
2nd dose	90	21 (30%)	50 (21.4%)	19 (28.8%)	
Booster dose	4	0	1(0.4%)	3 (4.6%)	
Moderna vaccine (mRNA-1273)					
1st dose	0	0	0	0	< 0.05
2nd dose	0	0	0	0	
Booster dose	25	22 (31.4%)	2 (0.9%)	1 (1.5%)	
Not sure of vaccination type	66	4 (5.7%)	48 (20.6%)	14 (21.2%)	–
Severity of symptoms					
Hospitalized	12	0	4 (1.7%)	8 (12.1%)	< 0.001
Non-hospitalized	357	70 (100%)	229 (98.3%)	58 (87.9%)	
Symptoms ≤ 3 months after recovery	59	25 (35.7%)	30 (12.9%)	4 (6.1%)	< 0.001
Symptoms 3–6 months after recovery	202	42 (60%)	108 (46.4%)	52 (78.8%)	< 0.001
Symptoms > 6 months after recovery	108	3 (4.3%)	95 (40.8%)	10 (17.5%)	< 0.001

### Description of COVID-19 Vaccination Among Study Individuals

**Fig. 2:**
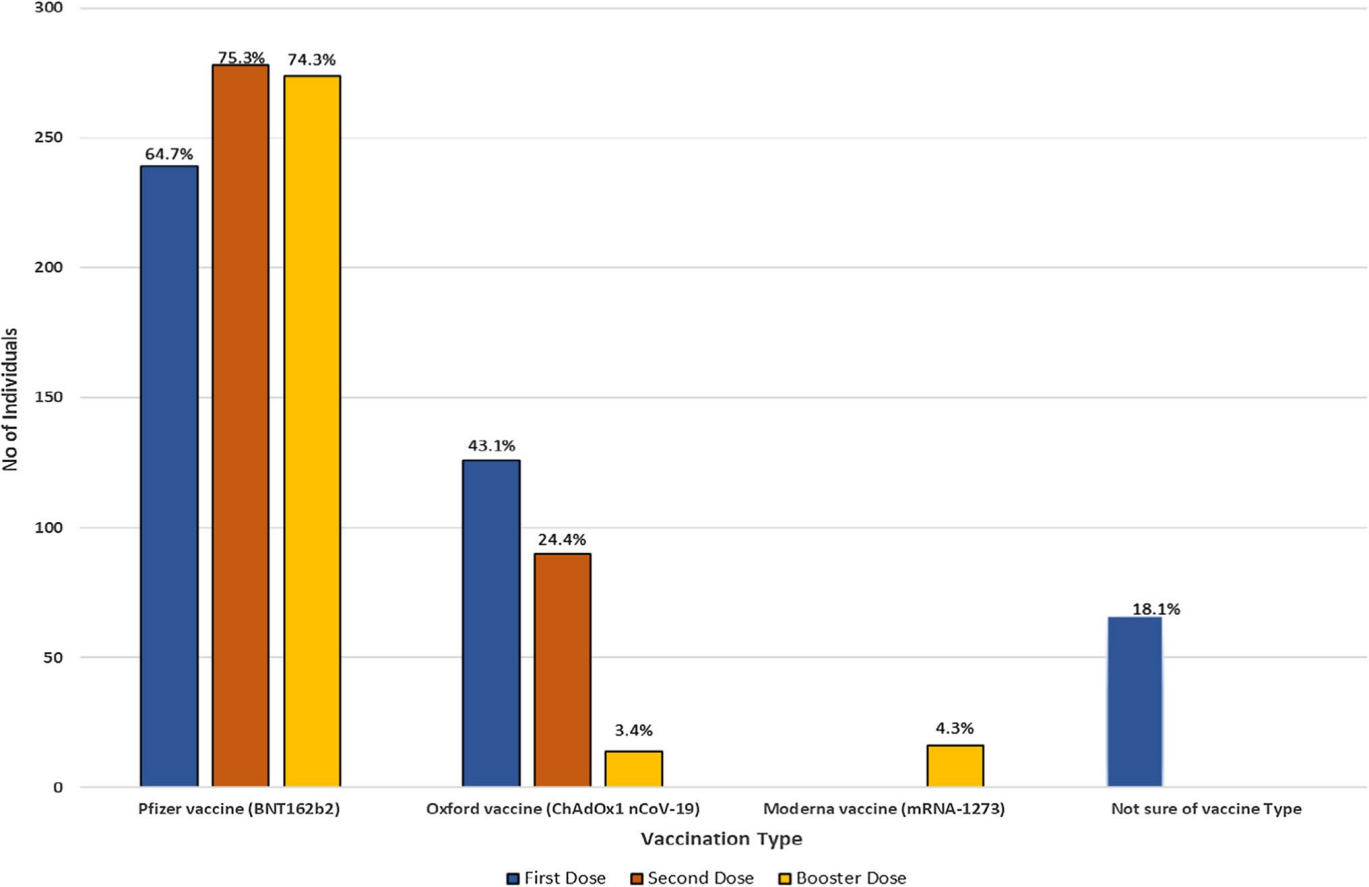
Distribution of vaccination among study patients

Of the total of 369 patients, the first dose was administered as early as December 2020 and 327 (88.8%) had a booster dose more than three months after the initial two doses and 41 (11.2%) had two does but not a booster dose. In terms of vaccine type distribution, 239 (64.7%), 278 (75.3%), and 274 (74.3%) patients received the Pfizer vaccine (BNT162b2) for the first, second, and booster doses, respectively. In addition, (43.1%), 90 (24.4%), and 4 (3.4%) had the Oxford-Astrazeneca vaccine (ChAdOx1 nCoV-19) for the first, second and third doses, respectively. A small portion, 16 (4.5%), received the Moderna vaccine (mRNA-1273) as the booster (third) dose. However, 66 (18.1%) of the respondents were uncertain about the type of booster dose they had received (Fig. 2). The time between the first COVID-19 vaccine and subsequent infection for the vaccinated group was at least three months.

### Characteristics of Post-COVID-19 Short- and Long-Term Symptoms

Post-COVID-19 symptoms occurred in 59 (16.1%) for ≤ 3 months, and 202 (54.8%) experienced persistent symptoms 3–6 months, and 108 (29.1%) reported symptoms lasting > 6 months. Of the included individuals, 237 (64.3%) had COVID-19 after receiving three doses of the vaccine, among them 234 (98.7%) were not hospitalized and were isolated at home and did not require oxygen supplement. However, only 3 (1.3%) were hospitalized for less than seven days and none required ICU admission. On the other hand, 102 (27.6%) were infected before vaccination and of them 8 7.8%) were hospitalized (P = 0.007); the remaining 30 (8.1%) of the total cases were unsure whether their infection occurred before or after vaccination.

**Fig. 3:**
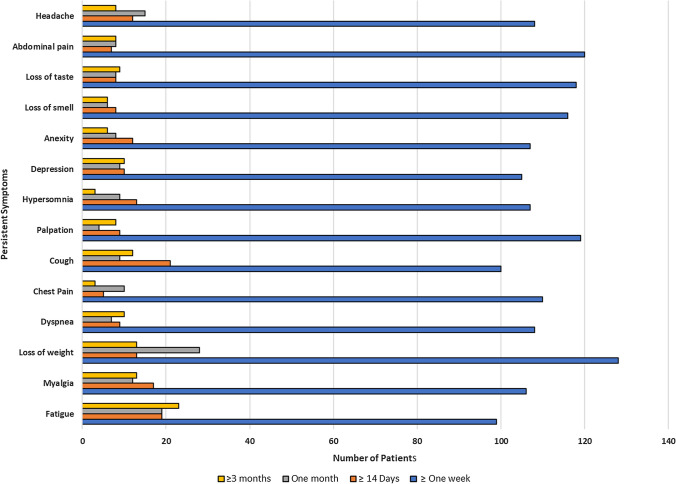
Number of patients with post-COVID-19 persistent symptoms of the included patients

Post-COVID-19 symptoms were weight loss (34.7%), abdominal pain (32.5%), palpitations (32.2%), loss of smell and taste (31.7%), and headaches (29.2%). Persistent symptoms lasting more than 14 days encompassed cough (5.7%), fatigue and myalgia (5.1%), and headaches and anxiety (3.2% each). In general, respiratory symptoms, such as cough, wheezing, and chest pain, persisted for one month in 20.3% of patients, alongside weight loss in 7.5% (Fig. 3).

Of the included patients, 108 (29.1%) reported symptoms lasting > 6 months and these symptoms included neuropsychiatric symptoms like concentration or memory deficits (7.8%) and headaches (2.1%). Anxiety and depression were reported by 5.9% of patients. Additional reported symptoms were dizziness (6.1%), itchiness (1.6%), fever (1.8%), and hair loss (1%).

In addition to post-COVID-19 symptoms, the survey assessed patients' perceptions of their general well-being after recovery. A majority (323; 87.5%) of the patients rated their health as good, 41 (11.1%) considered it fair, and 5 (1.4%) described their well-being as poor or terrible.

### Correlation of Post-COVID-19 Symptoms and the Relationship Between Vaccination and the Occurrence of Infection

Table 3 shows a multivariate analysis of the relation between vaccination and infection status and the development of post-COVID-19 symptoms. In terms of co-morbidities, the majority (82.3%) of those infected after full vaccination had no comorbidities, with a similar observation of 77.5% in those infected pre-vaccination. However, in the uncertain group, 53.3% had at least one co-morbidity and (46.7%) had no co-morbidities (p < 0.001).

**Table 3:** Multivariate analysis comparing the different groups in relation to vaccination status and the infection

Patients characteristics	Infection status in relation to the vaccination			
	Infected after receiving three doses of the vaccination (n = 237)	Infected prior to vaccination (n = 102)	Unsure of their infection in relation to the vaccination status (n = 30)	*P* value
Gender, n (%)				
Females, n (%)	126 (53.2%)	45 (44.1%)	6 (20%)	< 0.001
Males, n (%)	111(46.8%)	57 (55.8%)	24 (80%)	< 0.001
Nationality				
Saudi, n (%)	116 (48.9%)	79 (77.5%)	11 (36.6%)	< 0.001
Non-Saudi, n (%)	121(51.1%)	23 (22.5%)	19 (63.3%)	
Profession				
Healthcare workers	175 (73.8%)	49 (48%)	8 (26.6%)	< 0.001
Non-healthcare workers	60 (25.3%)	51 (50%)	18 (60%)	
Not working	2 (0.9%)	2 (1.9%)	4 (13.3%)	
Co-morbidities				
No–co morbidity	195 (82.3%)	79 (77.5%)	14 (46.7%)	< 0.001
≥ one co-morbidity	42 (17.7%)	23 (22.5%)	16 (53.3%)	
Pfizer vaccine (BNT162b2)				
1st dose	139 (73.8%)	90 (73.8%)	10 (73.8%)	< 0.05
2nd dose	96 (25.3%)	10 (25.3%)	19 (25.3%)	
Booster dose	2 (0.9%)	2 (0.9%)	1 (0.9%)	
Oxford vaccine (ChAdOx1 nCoV-19)				
1st dose	120 (50.6%)	67 (65.7%)	12 (73.8%)	< 0.05
2nd dose	114 (48.1%)	34 (33.3%)	16 (25.3%)	
Booster dose	3 (1.3%)	1 (0.9%)	2 (25.3%)	
Moderna vaccine (mRNA-1273)				
1st dose	0	0	0	< 0.05
2nd dose	0	0	0	
Booster dose	9 (3.8%)	7 (6.9%)	0	
Hospitalized	3 (1.3%)	8 (7.8%)	1 (3.3%)	< 0.001
Non-hospitalized	234 (98.7%)	94 (92.2%)	29 (96.7%)	
Symptoms ≤ 3 months after recovery	24 (10.1%)	12 (11.8%)	23 (76.7%)	< 0.001
Symptoms 3–6 months after recovery	123 (51.9%)	76 (74.5%)	3 (10%)	< 0.001
Symptoms > 6 months after recovery,	90 (38%)	14 (13.7%)	4 (13.3%)	< 0.001

Of the 237 patients who were infected after receiving three doses of the vaccine, 234 (98.7%) described their illness as mild to moderate (were isolated at home without the need to have oxygen supplementation); only 3 (1.3%) required admission for less than seven days; and none required ICU admission. On the other hand, of the 102 individuals infected before vaccination, 94 (92.2%) did not require hospitalization and 8 (7.8%) required hospitalization. These rates were higher among those infected prior to vaccination compared to those infected after receiving three doses of the vaccination (p-value < 0.00001) (Table 3).

Persistence of symptoms for > 3–6 months was more common in those who were infected prior to vaccination compared to those who were infected after vaccination (P < 0.001) (Table 3).

Table 4 presents the results of a regression analysis examining predictors of the development of post-COVID-19 symptoms. The analysis showed significant relationships between post-COVID-19 symptoms 3–6 months and the following predictors: gender, presence of co-morbidities, vaccination status, and severity of symptoms. Gender demonstrated a statistically significant association (95% CI = 0.12–0.94, P < 0.001) with females being less at risk compared to males. The presence of co-morbidities also significantly was associated with development of symptoms. Specifically, each additional co-morbidity was linked to a 0.23 increase in the odds of less recovery (95% CI = 0.15–0.31, P < 0.001). The vaccination status also significantly influenced recovery (95% CI = 0.33–0.73, P < 0.05). Lastly, severity of symptoms significantly affected recovery, there was an associated 0.33 decrease in the odds of recovery 3–6 months post-COVID-19 (95% CI = 0.05–0.65, P < 0.001) with each unit increase in symptom severity.

**Table 4:** A logistic regression analysis of the predictors of post-COVID-19 symptoms that persist 3–6 months

Predictors	Post COVID-19 recovery ≥ 3 months recovery		
	β coefficient	95% CI	P value
Gender (Female)	0.34	0.12, 0.94	< 0.001
Co-Morbidities (No comorbidities)	0.23	0.15, 0.31	< 0.001
Vaccination (before infection)	0.45	0.33, 0.73	< 0.05
Severity of symptoms (none severe)	0.33	0.05, 0.65	< 0.001

*β* Coefficient: regression coefficient, *CI* coefficient interval

## Discussion

This study explores variations in the probability of experiencing persistent symptoms post-COVID-19, and the study was conducted at the time when there were 826,009 confirmed cases of COVID-19 including 9,477 deaths in Saudi Arabia, from 3 January 2020 to 9 December 2022. We evaluated the occurrence and features of short and long term post-COVID-19 symptoms among 369 patients in Saudi Arabia.

The majority 237 (64.3%) had COVID-19 after receiving three doses of the vaccine, among them only 3 (1.3%) were hospitalized. These findings align with previous research suggesting that the majority of COVID-19 cases are mild to moderate in severity among vaccinated individuals and in the latter waves of the pandemic [15, 16]. The high number of hospitalizations (7.8%) among the individuals infected before vaccination contrasts sharply with the low hospitalization rate among those infected after receiving three doses of the vaccine (1.3%). The difference in hospitalization rates indicates the protective effect of vaccination against severe illness requiring hospital care, as was shown in a previous study from Saudi Arabia [17] and other studies.

The vaccination impact on individuals with existing long-COVID-19 symptoms is emerging and data showed changes in symptoms severity while others show no correlation between symptoms and vaccination status [18]. As known, low level of evidence (grade III, case-controls, cohort studies) indicates that vaccination before COVID-19 infection may reduce the risk of post-COVID-19 symptoms [18–20]. A previous study showed that vaccination before SARS-CoV-2 acute infection reduced long COVID-19 symptoms [18]. The vaccination impact on the development of long-COVID symptoms was variable in a scoping review of 16 studies [21]. An additional study showed that COVID-19 vaccination reduced long-COVID-19 symptoms at 6 weeks after illness onset from 79.1% among unvaccinated to 60.6% among vaccinated patients [22]. In a study of 1832 adults, the risk of symptoms > 28 days after SARS-CoV-2 symptoms was higher in unvaccinated at the time of infection [23] while Al-Aly et al. showed that two doses of COVID-19 vaccines would be more effective in reducing post-COVID-19 symptoms with certain vaccines better than others [24]. The current study showed that the rate of post-OCVID-19 was lower at 3–6 months among vaccinated individuals but was higher at > 6 months. This finding is intriguing and could be explained by recollection of symptoms, variability in severity of disease, or the occurrence of subsequent infections that had not been diagnosed. In one study, the occurrence of post-COVID-19 symptoms was 40.2% at 6 months after infection [25] and another study showed 29.6% had at least one symptom 6–12 months after testing compared to 13% of all test-negative patients [26]. However, these studies did not address the impact of vaccination on post-COVID-19 and further studies are waranted to explore this finding further.

Persistent symptoms included weight loss, abdominal pain, palpitations, loss of smell and taste, and headaches, while long-term symptoms encompassed cough, fatigue, myalgia, and neuropsychiatric symptoms. These findings are consistent with prior research reporting a wide range of persistent symptoms following COVID-19 infection [27–31]. The findings of this study showed that the majority of patients (61%) who reported symptoms after recovering from COVID-19 belonged to the 30–50 age group. This difference in symptom prevalence among different age groups was statistically significant (p < 0.001), in alignment with a previous study [32]. Additionally, other studies have reported the persistence of symptoms in vaccinated individuals as well [27] and age-related aspects of the findings echo similar results from other investigations. A recent meta-analysis emphasizes that middle-aged adults are more prone to post-COVID-19 symptoms [33]. This age group's vulnerability might be linked to a combination of immune response, pre-existing health conditions, and other socio-economic factors, though definitive reasons remain elusive [34, 35].

Interestingly, gender difference in post-COVID-19 observed in our study had also been reported, although we observed less long-COVID-19 among females. Previous studies reported gender differences in the occurrence of long COVID-19 as well as differences in the COVID-19 impact [24, 25]. However, the current study showed less impact on females and this finding might be related to the sample size or the recall of occurrence of symptoms. Females were more likely to report long COVID-19, a pattern that has been found in various cohorts and geographical regions [36]. However, the underlying biological or sociological reasons for this gender disparity need further exploration [37, 38]. The exact reasons why some individuals experience ongoing symptoms while others do not remain unclear, but it is believed to be associated with factors such as initial infection's severity, age, and presence of underlying health conditions [39]. In addition, this is a significant area for future research, as gendered insights into long COVID -19 could tailor public health measures and individual care.

The fact that 29% of participants in our study experienced persistent symptoms for more than six months highlights the potential long-term health implications of COVID-19. Other studies also reported the presence of neuropsychiatric symptoms, such as difficulties with concentration or memory, headaches, anxiety, and depression [23, 40]. These symptoms can have a significant impact on patients' quality of life and deserve further investigation [41–43]. Interestingly, despite the high prevalence of persistent symptoms, the majority of recovered patients (87.5%) rated their overall health as good. This suggests that, for many individuals, recovery from COVID-19 is associated with a generally positive perception of their health [44]. These findings are consistent with a study by Garrigues et al., which observed that most patients who had recovered from COVID-19 reported an overall improvement in their health status, with a significant decrease in symptom severity over time [45]. Additionally, another study found that more than half of the patients who had recovered from COVID-19 reported an enhancement in their quality of life, despite the presence of some persistent symptoms [46, 47]. These findings support the idea that many individuals maintain a positive outlook on their health following recovery from COVID-19, even in the presence of persistent symptoms. This aligns with the observation that the majority of recovered patients rated their health as good, indicating a generally positive perception of health post-recovery [48].

The lower hospitalization rates and the lack of ICU admissions in our study population, particularly among vaccinated individuals, align with global data reflecting the successful reduction in severe disease following vaccination [49]. Nevertheless, the persistence of symptoms in vaccinated individuals emphasizes the importance of a nuanced understanding of immunity, viral dynamics, and host factors. The precise mechanisms remain to be fully explored, and comprehensive studies are essential to inform clinical practice [50, 51]. Furthermore, the broad spectrum of persistent symptoms, including neuropsychiatric manifestations, aligns with the growing body of evidence that COVID-19 has multifaceted impacts on various systems [52, 53]. This suggests that a multidisciplinary approach to patient care is warranted, involving specialists in neurology, psychiatry, internal medicine, and other fields [54].

Our study contributes to the growing understanding of long COVID-19, offering valuable insights into the persistence of symptoms in relation to gender, age, vaccination status, and infection status. These findings should encourage a multidimensional and patient-centered approach to managing long COVID-19, integrating clinical care, research, and public health strategies. Continued collaboration between healthcare providers, researchers, and policymakers is essential to mitigate the ongoing and potentially long-lasting effects of this global health challenge.

It is important to acknowledge the limitations of our study. Firstly, the study sample was obtained through an online questionnaire distributed among various WhatsApp groups, which may introduce selection bias and limit the generalizability of our findings to the broader population. Secondly, our study design is cross-sectional, which makes it challenging to establish causal relationships between variables. Thirdly, the reliance on self-reported data in our study introduces the possibility of recall bias or the potential for participants to over or underreport their symptoms. Fourthly, the absence of a control group consisting of non-COVID-19-infected individuals makes it difficult to differentiate between post-recovery symptoms and symptoms related to comorbidities or other illnesses. Lastly, our study did not investigate the impact of vaccination on the severity of COVID-19 infection or hospitalization rates, which could have provided valuable insights into the effectiveness of the vaccine.

Despite these limitations, our study offers important insights into the persistence of symptoms in vaccinated individuals who have recovered from COVID-19. This information can contribute to the development of targeted interventions aimed at alleviating post-COVID-19 symptoms. It is crucial for healthcare providers to be aware of the potential for post-recovery symptoms in vaccinated individuals and to monitor their patients accordingly. Further research is needed to gain a better understanding of the underlying mechanisms behind persistent symptoms in vaccinated individuals and to identify effective interventions for long COVID.

## Data Availability

Data are available upon a reasonable request.

## Abbreviations

COVID-19: Coronavirus diseases 2019
95% CI: 95% Confidence interval
SARS-CoV-2: Severe acute respiratory syndrome coronavirus 2
PASC: Post-acute sequelae of SARS-CoV-2 infection
